# A complicated case of myocardial infarction with nonobstructive coronary arteries with an underlying pheochromocytoma: a case report

**DOI:** 10.1186/s40885-021-00189-9

**Published:** 2022-02-01

**Authors:** Sodam Jung, In Sook Kang

**Affiliations:** Division of Cardiology, Department of Internal Medicine, Ewha Womans University Mokdong Hospital, Seoul, Republic of Korea

**Keywords:** Pheochromocytoma, Myocardial infarction, Cerebral hemorrhage, Catecholamine

## Abstract

**Background:**

The signs and symptoms of pheochromocytoma can imitate those of many other diseases, which may result in confusion. Therefore, diagnosing and treating secondary hypertension due to pheochromocytoma in deteriorating patients becomes challenging.

**Case presentation:**

A 63-year-old female patient presented to the emergency room with severe and progressive nausea. The initial diagnosis was an acute myocardial infarction based on ST-segment depression on electrocardiogram and elevated cardiac markers. Elective coronary angiography revealed nonobstructive coronary arteries. However, she suffered from a complicated clinical course for several weeks during her life-or-death crisis. She was subsequently diagnosed with a cerebral hemorrhage and a pheochromocytoma. It is unclear whether her initial presentation was due to the neurogenic stunned myocardium caused by a cerebral hemorrhage or type 2 myocardial infarction caused by a pheochromocytoma, or both. However, this case showed the significance of accurately diagnosing and treating underlying causes in patients presenting with myocardial infarction with nonobstructive coronary arteries. Early diagnosis and treatment of the pheochromocytoma may have prevented the complications experienced by the patient.

**Conclusions:**

A catecholamine surge and blood pressure fluctuation caused severe complications. When a patient presents with an unusual clinical presentation, secondary hypertension due to pheochromocytoma should be suspected.

## Background

Early angiography studies in the 1980s have detected occluded arteries in 90 and 30% of cases with acute ST-segment elevation myocardial infarction (STEMI) and non-ST-segment elevation myocardial infarction (NSTEMI), respectively [1, 2]. These studies have also demonstrated significant obstructive coronary artery disease in 97% of cases of both STEMI and NSTEMI. Therefore, it is important to recognize atherosclerotic coronary artery disease.

Recent multi-center registries have demonstrated that up to 10% of patients with myocardial infarction (MI) do not have coronary artery obstruction [3]. Thus, the term myocardial infarction with nonobstructive coronary arteries (MINOCA) was introduced [4]. Since conventional MI was thought to be caused only by pronounced coronary artery obstruction, physicians tended to consider angiography only briefly. Nowadays, physicians look more thoroughly to find small causes that can be easily overlooked, such as minimal intraluminal dissection, vasospasm, and other minimal intracoronary diseases that may cause MI in MINOCA. Hence, there is a higher probability of finding the cause of MINOCA and treating it appropriately than before [5]. MINOCA is diagnosed after excluding other causes of elevated cardiac biomarkers, such as pulmonary thromboembolism and acute pericarditis [6]. Thus, efforts should be made to detect non-coronary causes.

Acute MI is diagnosed based on ischemic changes seen on electrocardiograms (EKG), cardiac biomarker elevation and ischemic symptoms with a duration of at least 20 min. Additional symptoms with exertion or at rest include chest, epigastric, jaw, and upper extremity discomfort. The discomfort is often unspecific and accompanied by dyspnea, diaphoresis, nausea, or syncope [7]. Therefore, complaints of nausea, vomiting, and epigastric discomfort combined with ischemic changes on an EKG or elevated cardiac biomarkers lead to a diagnosis of MI. Treatment is generally initiated immediately. However, EKG changes and cardiac marker elevation may appear in other diseases, such as pericarditis, ascending aortic disease, pulmonary embolism, hyperkalemia, and intracranial hemorrhage (ICH) [8].

The ST-T segment changes on EKG, which are typically seen in MI, and symptoms, such as nausea and vomiting, which can occur in right coronary artery disease, can also be present in an ICH [9]. However, the treatment strategies for these conditions are entirely different.

Another condition is pheochromocytoma, which reportedly may cause MI, heart failure, cerebral hemorrhage, fever, and metabolic acidosis. Only a few cases have involved cerebral hemorrhage, and only a small number of patients survived [10, 11].

Here, we present a complicated case of pheochromocytoma with vague epigastric discomfort and vomiting.

## Case presentation

**Fig. 1:**
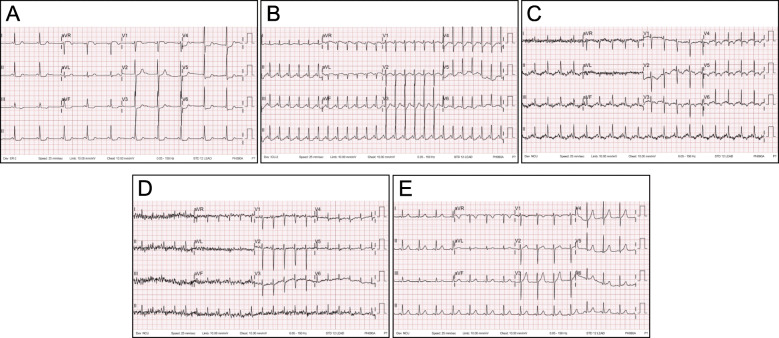
Electrocardiogram obtained at the patient’s first visit to the emergency room. The ST depression observed at admission (**A**) persisted after coronary angiography (**B**), but partially improved after brain surgery (hospital day 3), wherein T-wave inversion and tachycardia were observed (**C**). After 4 days (hospital day 7), the T-wave inversion normalized but the tachycardia persisted (**D**). Prior to starting an alpha-blocker for pheochromocytoma (hospital day 11), ST depression showed improvement with a sinus rhythm (hospital day 22) (**E**). After that, a normal sinus rhythm was maintained, and electrocardiography was not performed daily

**Fig. 2:**
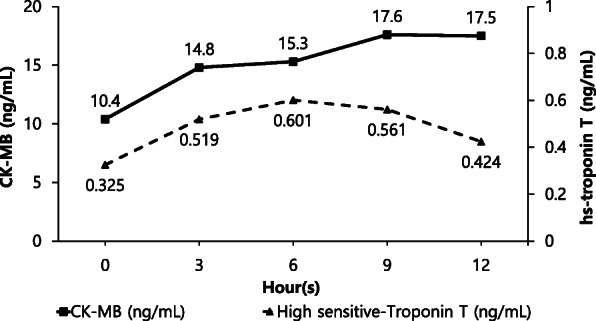
Cardiac biomarker during the first 12 h. Creatine kinase-MB (CK-MB) level remained elevated until 12 h after admission. On the other hand, hs-troponin-T level was elevated for the first 6 h after admission before decreasing

A 63-year-old female patient with hypothyroidism presented to our hospital with continuous nausea and five episodes of vomiting. Her complaints began after ingesting steak and a glass of wine 9 h prior to admission. Her supine blood pressure was 184/75 mmHg, and her heart rate was 68 bpm. The patient occasionally did visit a local clinic due to her high blood pressure, but upon subsequent measurements, her blood pressure was found to be normal, and therefore, she was not treated. EKG showed ST depression in leads II, III, aVF, and V4, 5, and 6 (Fig. 1A). Creatine kinase-MB and high-sensitivity (hs)-troponin T levels were 10.4 ng/mL (reference range, 0–0.5 ng/mL) and 0.325 ng/mL (reference range, > 0.014), respectively. After 3 h, her hs-troponin-T levels rose to 0.519 ng/mL (Fig. 2); therefore, she was diagnosed with NSTEMI and received intravenous unfractionated heparin and oral dual antiplatelet therapy. She was scheduled for an early coronary angiography (CAG). Transthoracic echocardiography showed inferior wall hypokinesia with a normal ejection fraction.

**Fig. 3:**
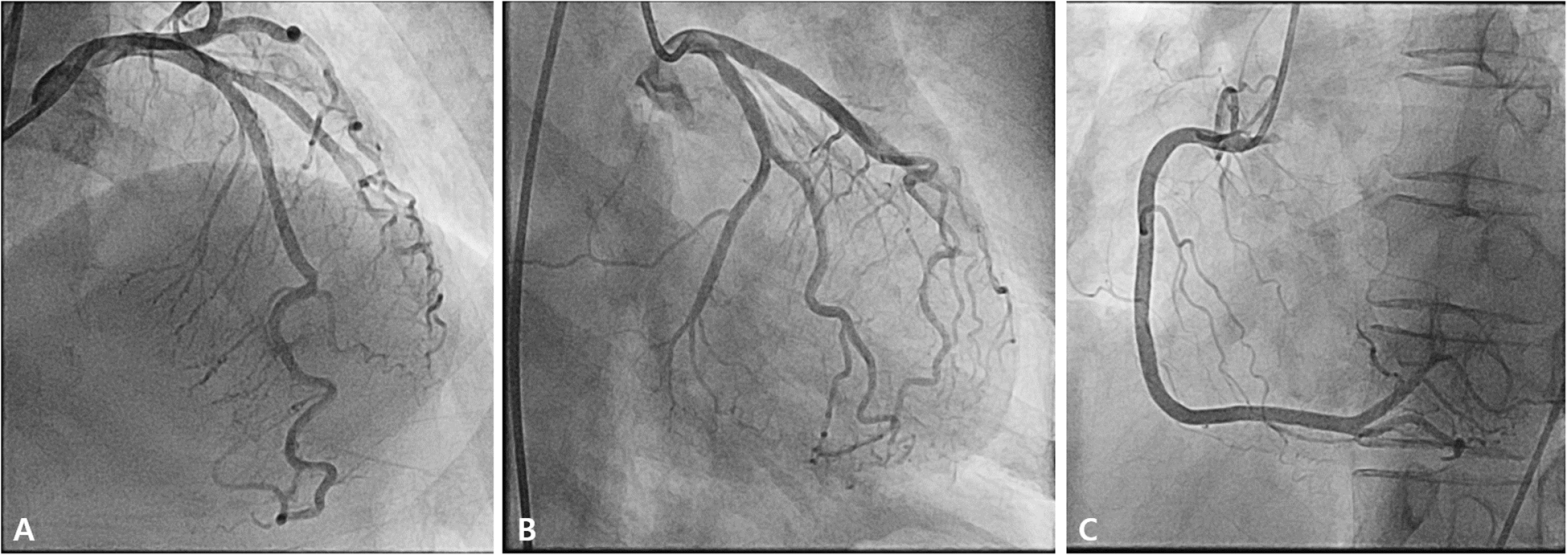
Coronary angiography 12 h after admission. Coronary angiography showed no significant luminal narrowing in both coronary arteries. **A** antero-posterior cranial view of left coronary angiography, (**B)** right anterior oblique caudal view of left coronary angiography, C: left anterior oblique view of right coronary angiography

**Fig. 4:**
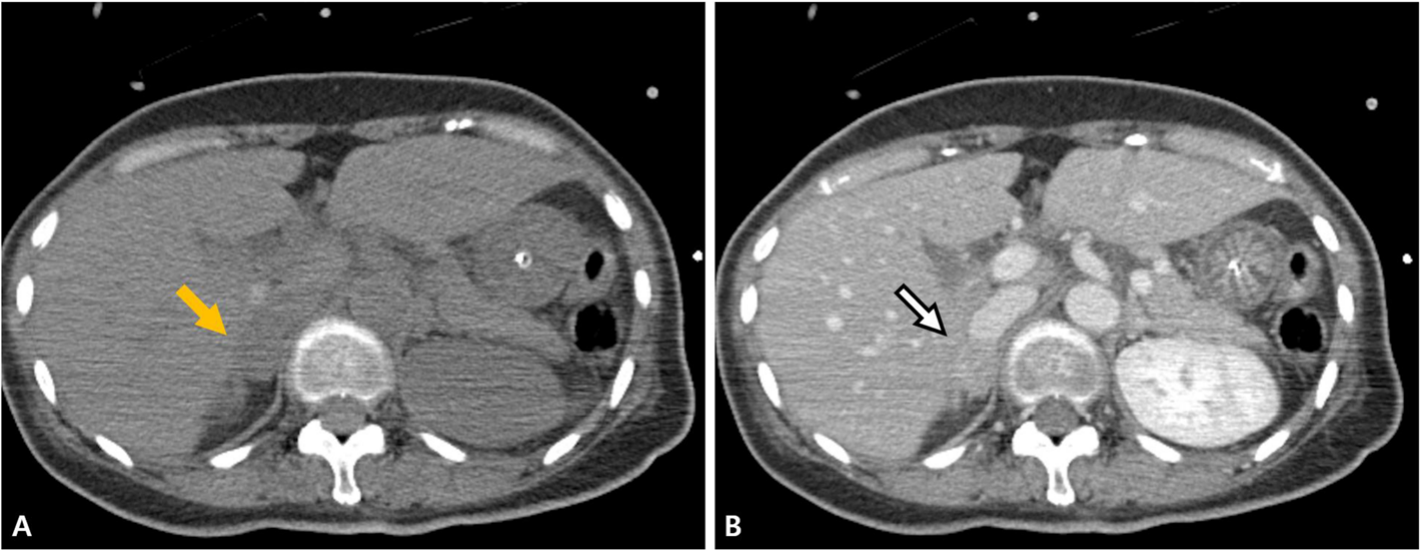
Abdominopelvic computed tomography (CT). **A** Pre-contrast abdominopelvic CT scan demonstrated a 1.7-cm-sized nodule (arrow), (**B**) contrast-enhanced CT showed enhancement (arrow) suggestive of an adenoma and confirmed by excisional biopsy with adrenalectomy

**Fig. 5:**
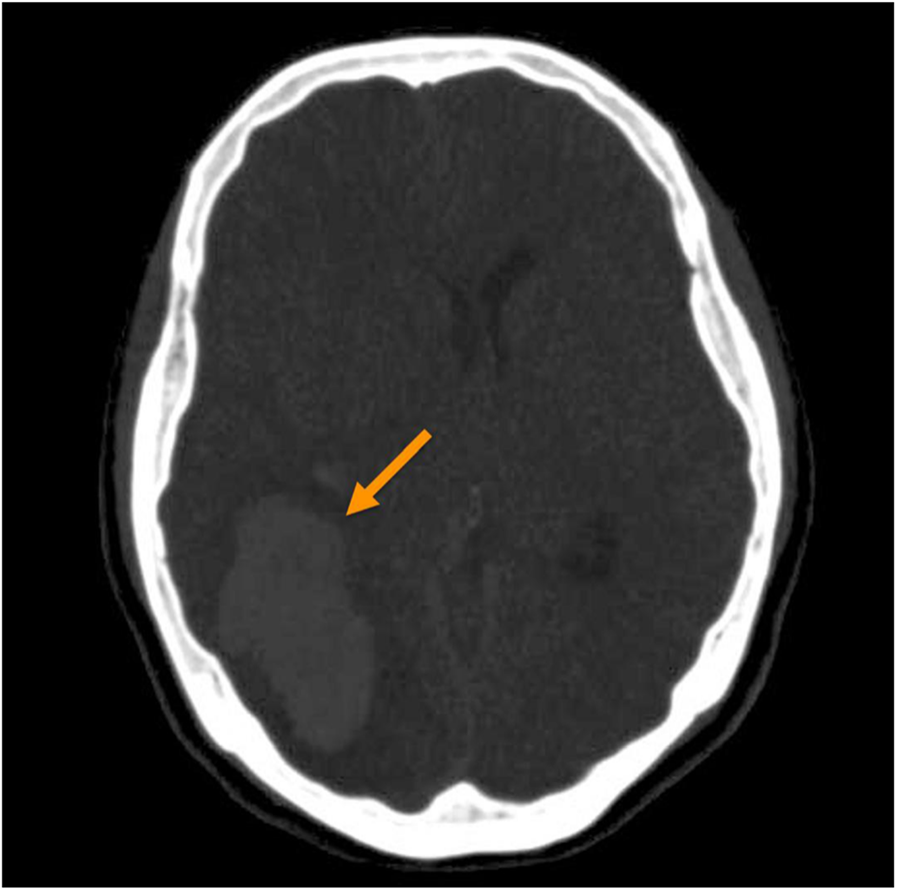
Non-enhanced brain computed tomography (CT). Brain CT showed an acute intracerebral hemorrhage (arrow) with perilesional edema at the parietotemporooccipital lobe. An acute intraventricular hemorrhage causing a midline shift to the left was also noted

Twelve hours after admission, CAG was performed, and the findings were unremarkable, except for mild myocardial bridging at the mid portion of the left anterior descending artery (Fig. 3). The patient experienced persistent nausea and dizziness, both during and after the angiography; therefore, dual antiplatelet therapy and heparin treatment were immediately discontinued. Abdominopelvic computed tomography (CT) was performed to exclude gastrointestinal causes. It demonstrated a patent gallbladder, stomach, and intestines. However, a 1.7 cm enhanced nodule was detected on the right adrenal gland, suggesting an adenoma (Fig. 4). Further physical examination revealed a newly developed gaze palsy. Brain CT revealed an acute ICH with perilesional edema in the parietotemporooccipital lobe and an acute intraventricular hemorrhage, causing a midline shift to the left (Fig. 5). After undergoing emergency craniotomy with hematoma evacuation in the intensive care unit (ICU), her cardiac biomarkers and EKG gradually normalized. During the 1-month recovery period, she developed pneumonia and deep vein thrombosis, which extended her ICU stay. Furthermore, she required treatment for high blood pressure (systolic blood pressure up to 170 mmHg) since her blood pressure was uncontrolled despite combination therapy with nimodipine (60 mg six times daily), amlodipine (5 mg twice a day), candesartan (16 mg daily), carvedilol (12.5 mg twice daily), and doxazosin (4 mg once daily), with continuous intravenous nicardipine infusion (up to 40 mg per hour). Laboratory test results performed for the analysis of the uncontrolled hypertension and adrenal adenoma showed increased urinary catecholamines level (epinephrine > 230 mcg/day, reference value < 80 mcg/day; norepinephrine 625.33 mcg/day, reference value < 80 mcg/day; dopamine > 919.75 mcg/day, reference values 65–400 mcg/day; metanephrine > 7.5 mcg/day, reference value < 0.71 mcg/day). Consequently, the patient was diagnosed with pheochromocytoma. She received phenoxybenzamine (30 mg/day for 2 weeks) and underwent an adrenalectomy. A biopsy confirmed the diagnosis of pheochromocytoma. After the surgery, amlodipine 5 mg once daily and carvedilol 12.5 mg twice daily successfully controlled her blood pressure (130/75 mmHg). Urine and plasma catecholamine levels normalized. Figure 1A–E shows the improved ST depression over time. Inferior wall hypokinesia on echocardiography was sustained. She is now clinically stable and alert.

## Discussion

The patient was diagnosed with ICH. After cranial decompression surgery, symptoms such as nausea and chest tightness, and neurologic abnormalities improved. However, her hypertension persisted. She was additionally diagnosed with pheochromocytoma and treated by adrenalectomy. Finally, she was discharged and remained clinically stable.

Her initial symptoms were nausea and vomiting, which are not typical but are common to ICH and MI. Her initial diagnosis in the emergency room (ER) was NSTEMI, based on the cardiac marker elevation and ST depression seen on the EKG. Early CAG was performed to determine the cause of MI and coronary artery stenosis. CAG showed no apparent coronary artery stenosis.

There is an increased interest in the diagnosis of MINOCA. MINOCA is defined by the 2016 European Society of Cardiology position paper [6] as an MI without obstructive coronary artery disease and no other clinical findings suggestive of alternative causes for the elevated cardiac biomarkers. The prognosis of MINOCA is not favorable compared to the conventional causes of MI. In a nationwide prospective study of the Korean Acute Myocardial Infarction- National Institutes of Health registry [12], MINOCA and MI with coronary artery occlusive disease had similar frequencies of in-hospital events, rates of mortality, and recurrent MI within 2 years. Thus, after ruling out other causes, clinicians should identify the etiology of MINOCA, such as plaque disruption, coronary artery dissection, epicardial or microvascular spasm, and coronary thromboembolism [6, 13]. When we saw this patient’s nonobstructive coronary arteries, we considered performing the ergonovine provocation test to rule out vasospasm, but we decided to defer this test due to her unstable condition. In our case, MINOCA may have been suspected upon ruling out non-cardiac causes of myocardial damage. The decreasing biomarkers prior to CAG suggests that the MI was improving. It is assumed that the myocardial damage improved as the coronary artery spasms decreased when the catecholamine concentration fluctuated. MINOCA is a working diagnosis and refers to an MI with coronary artery problems but without significant occlusion on CAG [13]. It corresponds to a diagnosis that highlights the need to ascertain the causes of coronary vasospasm and dissection. A diagnosis of MINOCA is excluded if there are other specific causes for the MI, such as ICH and pheochromocytoma surge, as in our case. Since we were not aware of the existence of an ICH until after a brain CT was performed and after a CAG confirmed it, we suspected MINOCA immediately after the CAG. As ICH and pheochromocytoma were eventually confirmed, this patient does not fit the criteria for MINOCA. We attempted to look at the non-cardiac possibilities by performing abdominopelvic CT, brain CT, and additional physical examinations. On the other hand, a cardiac MRI is not only a good option for diagnosing MINOCA but also has the added advantage of helping predict the prognosis by identifying the extent of myocardial damage [14]. However, in our patient, since the cardiac biomarkers improved, a cardiac MRI was not performed as there was no evidence of deteriorating cardiac function.

A detailed evaluation revealed an ICH and a pheochromocytoma as the causes of raised cardiac biomarkers and other symptoms. Neurogenic stunned myocardium (NSM) is a cardiac complication that occurs after a neurologic event due to the dysregulation of the autonomic nervous system [15]. NSM presents with ischemic EKG changes, elevated cardiac markers, and ventricular wall motion abnormalities, similarly to MI. The potential mechanism of NSM involves a catecholamine surge after damage to the regions of the brain governing the autonomic system. Meanwhile, MI is due to coronary artery blockage. Significant coronary artery obstruction is absent in NSM [16]. Recent cases of ICH mimicking STEMI have been reported [17]. The signs and symptoms of MI and ICH, such as nausea, vomiting, and syncope, are ambiguous and may confuse the diagnosis.

There are several reported cases with similar or related pathophysiology to our case. In addition, cocaine-induced MI and stress-induced cardiomyopathy (SCMP) [18] presenting similarly to a myocardial injury as a result of an underlying ICH [17] and pheochromocytoma [19, 20], respectively, have also been reported.

A characteristic feature seen in our patient which differs from other similar cases is that our patient who developed an acute MI had an underlying ICH and a pheochromocytoma. Cases of NSM that appear as a part of the clinical course of ICH, and MI due to pheochromocytoma have been reported as mentioned above [16, 17, 19, 20]; however, there were no cases in which the two conditions appeared together. MI resulting from an ICH and a pheochromocytoma have in common that both are a result of a catecholamine rush. The order of the myocardial injury in this case is unclear (Fig. 7). Considering the clinical course, one of the two hypotheses is that the pheochromocytoma crisis occurred after the wine intake, and the ICH developed as a result. In this case, the myocardial injury confirmed upon admission to the ER was thought to be related to the catecholamines derived from the pheochromocytoma or ICH. Another hypothesis is that only the ICH occurred initially, and the pheochromocytoma crises developed after the surgery. In this case, it is presumed that the catecholamine release, which is one of the mechanisms of NSM, caused the myocardial injury.

Cocaine-induced MI appears as a normal coronary artery, vasospasm, or a coronary artery thrombosis [21]. Supply-demand mismatch caused by the catecholamine rush and coronary artery spasm caused by the cocaine’s adrenergic reuptake blockade are similar to our case, but the difference is that a thrombotic component [22] is likely to coexist in cases of cocaine abuse.

In general, SCMP or catecholamine associated CMP (NSM, cocaine, pheochromocytoma) shows regional wall motion abnormalities that do not fit well with coronary artery territories [13]. This is also a good indication when diagnosing SCMP. However, early echocardiography in our patient showed hypokinesia of the inferior wall. A possible explanation for this is, first, that the cases of stress-induced CMP appearing in a single vessel territory are rare but have been reported [23]. Although the diagnosis of SCMP is excluded in the presence of a pheochromocytoma or an NSM, it is also accepted that a catecholamine surge plays a part in the pathophysiology of SCMP [24]. Therefore, if an SCMP appears as a single vessel territory, a single vessel territory hypokinesia is possible even in the case of a pheochromocytoma or an NSM. On the other hand, a follow-up echocardiogram performed 3 months later showed a base to mid inferior wall hypokinesia. In the CAG, despite an inferior wall hypokinesia, the right coronary artery was normal without stenosis. The cerebral hemorrhage was resolved and the pheochromocytoma was resected.

In our case, the patient’s nausea persisted, and further evaluation identified a lateral gaze palsy. If brain CT was performed early in the ER, heparin and dual antiplatelet therapy, which increase the risk of bleeding, could be avoided. Also, the surgery for the cerebral hemorrhage could have been performed earlier. However, when our patient presented to the ER, her complaints did not include any specific neurological symptoms, such as headache or decreased consciousness. Thus, it was difficult to ascertain a neurological diagnosis.

Pheochromocytoma may have caused the cerebral hemorrhage and the abrupt elevation of blood pressure that followed. Pheochromocytoma is a rare tumor derived from the chromaffin cells. It is characterized by the production of catecholamines and other neuropeptides [25]. The clinical presentation of pheochromocytoma differs from blood pressure fluctuations due to an MI. The catecholamine surge induces vascular constriction and myocardial inotrope effects, which result in myocardial damage [26]. Pheochromocytoma is aggravated by consuming foods or beverages, such as wine, that are rich in tyramine [27]. At admission, her heart rate was 68 bpm, which is lower than what we anticipated considering pheochromocytoma. Moreover, blood pressure and pulse rate may fluctuate even during a pheochromocytoma crisis. Therefore, one hypothesis is that the patient developed a cerebral hemorrhage and cardiomyopathy initially during the crisis, and upon arrival to the hospital, the catecholamine surge had decreased temporarily. After the surgical treatment of the cerebral hemorrhage and the treatment of hypothermia, it is presumed that the adrenal surge was not revealed, and hypertension due to the pheochromocytoma was revealed again. Another hypothesis is that the pheochromocytoma was initially not a problem, but after the surgical treatment of the cerebral hemorrhage, it was stimulated by a stressful condition resulting in elevated blood pressure.

**Fig. 6:**
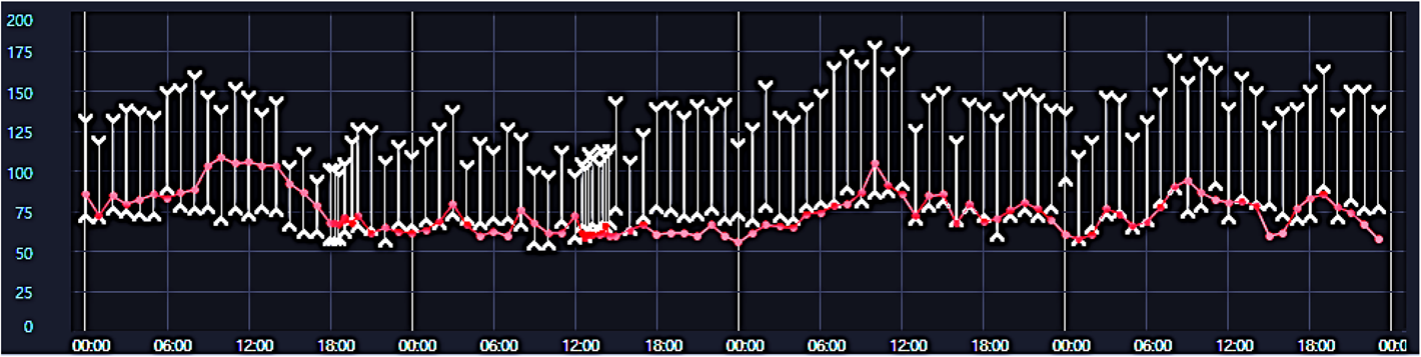
Fluctuation of blood pressure and pulse rate. The patient’s blood pressure and pulse rate were generally parallel before the diagnosis of pheochromocytoma from hospital day 15 to 18. Red line, pulse rate; white line with wedges, upper wedge-systolic blood pressure; lower wedge, diastolic blood pressure

Both hypotheses suggest that the postoperative hypertension is due to the pheochromocytoma. This is based on the fluctuations in the patient’s blood pressure and pulse rate were generally parallel post-surgery (Fig. 6). Moreover, before visiting the ER, the patient had experienced increased blood pressure at home, but this was not documented in the hospital setting. This intermittent blood pressure elevation was likely a manifestation of the pheochromocytoma.

**Fig. 7:**
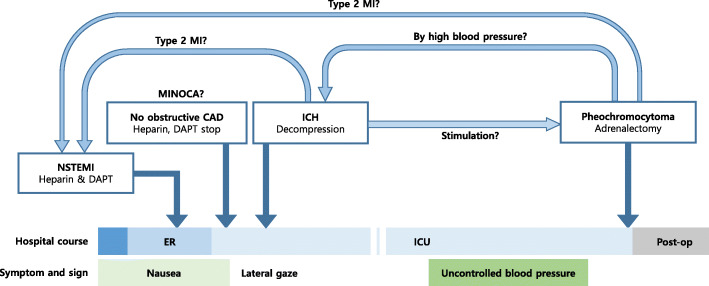
Clinical course. The patient initially presented as a non-ST elevation myocardial infarction. However, further evaluation revealed intracranial hemorrhage (ICH) and pheochromocytoma. Two scenarios are possible: stimuli may have triggered pheochromocytoma, resulting in a catecholamine crisis, or a spontaneous ICH resulted in a neurogenic stunned myocardium syndrome (elevated cardiac markers). ICH and decompression surgery may have resulted in uncontrolled increases in blood pressure due to pheochromocytoma. MI: myocardial infarction, MINOCA: myocardial infarction with nonobstructive coronary arteries, CAD: coronary artery disease, NSTEMI: non-ST elevation myocardial infarction, DAPT: dual-antiplatelet therapy, ER: emergency room, ICU: intensive care unit

After the ICH treatment, her cardiac marker values normalized. Moreover, the resection of the pheochromocytoma reduced the number of anti-hypertensives required. Therefore, we assumed a catecholamine surge triggered by ingesting a tyramine-rich beverage caused paroxysmal hypertension and resulted in an ICH. Myocardial damage occurred as a direct effect of the catecholamine surge associated with pheochromocytoma. NSM from ICH also induced myocardial damage in this patient (Fig. 7).

## Conclusions

We present a rare case of ICH accompanied by pheochromocytoma, which initially presented as an MI. Type 2 MI and MINOCA have a poor prognosis, similar to type 1 MI, and require immediate treatment. However, their treatments may worsen ICH. Therefore, formulating a differential diagnosis is crucial for proper treatment. This case showed that other causes of myocardial damage, such as a catecholamine surge, should be suspected when myocardial ischemic signs and vague symptoms are present. The early diagnosis and treatment of pheochromocytoma is critical.

## Data Availability

Not applicable.

## Abbreviations

STEMI: Acute ST-segment elevation myocardial infarction
NSTEMI: Non-ST-segment elevation myocardial infarction
MI: myocardial infarction
MINOCA: Myocardial infarction with nonobstructive coronary arteries
EKG: Electrocardiogram
ICH: Intracranial hemorrhage
CAG: Coronary angiography
CT: Computed tomography
ICU: Intensive care unit
NSM: Neurogenic stunned myocardium
SCMP: Stress-induced cardiomyopathy.

## References

[1] DeWood MA, Spores J, Notske R, Mouser LT, Burroughs R, Golden MS (1980). Prevalence of total coronary occlusion during the early hours of transmural myocardial infarction. N Engl J Med.

[2] DeWood MA, Stifter WF, Simpson CS, Spores J, Eugster GS, Judge TP (1986). Coronary arteriographic findings soon after non-Q-wave myocardial infarction. N Engl J Med.

[3] Gehrie ER, Reynolds HR, Chen AY, Neelon BH, Roe MT, Gibler WB, Ohman EM, Newby LK, Peterson ED, Hochman JS (2009). Characterization and outcomes of women and men with non-ST-segment elevation myocardial infarction and nonobstructive coronary artery disease: results from the can rapid risk stratification of unstable angina patients suppress adverse outcomes with early implementation of the ACC/AHA guidelines (CRUSADE) quality improvement initiative. Am Heart J.

[4] Beltrame JF (2013). Assessing patients with myocardial infarction and nonobstructed coronary arteries (MINOCA). J Intern Med.

[5] Tamis-Holland JE, Jneid H (2018). Myocardial infarction with nonobstructive coronary arteries (MINOCA): it’s time to face reality!. J Am Heart Assoc.

[6] Agewall S, Beltrame JF, Reynolds HR, Niessner A, Rosano G, Caforio AL (2017). ESC working group position paper on myocardial infarction with non-obstructive coronary arteries. Eur Heart J.

[7] Thygesen K, Alpert JS, Jaffe AS, Chaitman BR, Bax JJ, Morrow DA, White HD, Thygesen K, Alpert JS, Jaffe AS, Chaitman BR, Bax JJ, Morrow DA, White HD, Mickley H, Crea F, van de Werf F, Bucciarelli-Ducci C, Katus HA, Pinto FJ, Antman EM, Hamm CW, de Caterina R, Januzzi JL, Apple FS, Alonso Garcia MA, Underwood SR, Canty JM, Lyon AR, Devereaux PJ, Zamorano JL, Lindahl B, Weintraub WS, Newby LK, Virmani R, Vranckx P, Cutlip D, Gibbons RJ, Smith SC, Atar D, Luepker RV, Robertson RM, Bonow RO, Steg PG, O’Gara PT, Fox KAA, Hasdai D, Aboyans V, Achenbach S, Agewall S, Alexander T, Avezum A, Barbato E, Bassand JP, Bates E, Bittl JA, Breithardt G, Bueno H, Bugiardini R, Cohen MG, Dangas G, de Lemos JA, Delgado V, Filippatos G, Fry E, Granger CB, Halvorsen S, Hlatky MA, Ibanez B, James S, Kastrati A, Leclercq C, Mahaffey KW, Mehta L, Müller C, Patrono C, Piepoli MF, Piñeiro D, Roffi M, Rubboli A, Sharma S, Simpson IA, Tendera M, Valgimigli M, van der Wal AC, Windecker S, Chettibi M, Hayrapetyan H, Roithinger FX, Aliyev F, Sujayeva V, Claeys MJ, Smajić E, Kala P, Iversen KK, el Hefny E, Marandi T, Porela P, Antov S, Gilard M, Blankenberg S, Davlouros P, Gudnason T, Alcalai R, Colivicchi F, Elezi S, Baitova G, Zakke I, Gustiene O, Beissel J, Dingli P, Grosu A, Damman P, Juliebø V, Legutko J, Morais J, Tatu-Chitoiu G, Yakovlev A, Zavatta M, Nedeljkovic M, Radsel P, Sionis A, Jemberg T, Müller C, Abid L, Abaci A, Parkhomenko A, Corbett S, ESC Scientific Document Group (2019). Fourth universal definition of myocardial infarction (2018). Eur Heart J.

[8] Korff S, Katus HA, Giannitsis E (2006). Differential diagnosis of elevated troponins. Heart..

[9] Zaroff JG, Rordorf GA, Newell JB, Ogilvy CS, Levinson JR (1999). Cardiac outcome in patients with subarachnoid hemorrhage and electrocardiographic abnormalities. Neurosurgery..

[10] Scardigli K, Biller J, Brooks MH, Cespedes LE, Posniak HV (1985). Pontine hemorrhage in a patient with pheochromocytoma. Arch Intern Med.

[11] Mizukami H, Hara S, Kobayashi M, Mori S, Kuriiwa F, Fukunaga T (2013). An autopsy case of bilateral adrenal pheochromocytoma-associated cerebral hemorrhage. Leg Med (Tokyo).

[12] Choo EH, Chang K, Lee KY, Lee D, Kim JG, Ahn Y, Kim YJ, Chae SC, Cho MC, Kim CJ, Kim HS, Jeong MH, KAMIR‐NIH Investigators (2019). Prognosis and predictors of mortality in patients suffering myocardial infarction with non-obstructive coronary arteries. J Am Heart Assoc.

[13] Tamis-Holland JE, Jneid H, Reynolds HR, Agewall S, Brilakis ES, Brown TM, Lerman A, Cushman M, Kumbhani DJ, Arslanian-Engoren C, Bolger AF, Beltrame JF, American Heart Association Interventional Cardiovascular Care Committee of the Council on Clinical Cardiology; Council on Cardiovascular and Stroke Nursing; Council on Epidemiology and Prevention; and Council on Quality of Care and Outcomes Research (2019). Contemporary diagnosis and management of patients with myocardial infarction in the absence of obstructive coronary artery disease: a scientific statement from the American Heart Association. Circulation..

[14] Collste O, Sörensson P, Frick M, Agewall S, Daniel M, Henareh L, Ekenbäck C, Eurenius L, Guiron C, Jernberg T, Hofman-Bang C, Malmqvist K, Nagy E, Arheden H, Tornvall P (2013). Myocardial infarction with normal coronary arteries is common and associated with normal findings on cardiovascular magnetic resonance imaging: results from the Stockholm myocardial infarction with Normal coronaries study. J Intern Med.

[15] Chen Z, Venkat P, Seyfried D, Chopp M, Yan T, Chen J (2017). Brain-heart interaction: cardiac complications after stroke. Circ Res.

[16] Biso S, Wongrakpanich S, Agrawal A, Yadlapati S, Kishlyansky M, Figueredo V (2017). A review of neurogenic stunned myocardium. Cardiovasc Psychiatry Neurol.

[17] Park I, Kim YJ, Ahn S, Sohn CH, Seo DW, Kim WY (2015). Subarachnoid hemorrhage mimicking ST-segment elevation myocardial infarction after return of spontaneous circulation. Clin Exp Emerg Med.

[18] McCord J, Jneid H, Hollander JE, de Lemos JA, Cercek B, Hsue P, Gibler WB, Ohman EM, Drew B, Philippides G, Newby LK, American Heart Association Acute Cardiac Care Committee of the Council on Clinical Cardiology (2008). Management of cocaine-associated chest pain and myocardial infarction: a scientific statement from the American Heart Association acute cardiac Care Committee of the Council on clinical cardiology. Circulation..

[19] Prejbisz A, Lenders JW, Eisenhofer G, Januszewicz A (2011). Cardiovascular manifestations of phaeochromocytoma. J Hypertens.

[20] Menke-van der Houven van Oordt CW, Twickler TB, van Asperdt FG, Ackermans P, Timmers HJ, Hermus AR (2007). Pheochromocytoma mimicking an acute myocardial infarction. Neth Heart J.

[21] Schwartz BG, Rezkalla S, Kloner RA (2010). Cardiovascular effects of cocaine. Circulation..

[22] Heesch CM, Wilhelm CR, Ristich J, Adnane J, Bontempo FA, Wagner WR (2000). Cocaine activates platelets and increases the formation of circulating platelet containing microaggregates in humans. Heart..

[23] Ghadri JR, Wittstein IS, Prasad A, Sharkey S, Dote K, Akashi YJ, Cammann VL, Crea F, Galiuto L, Desmet W, Yoshida T, Manfredini R, Eitel I, Kosuge M, Nef HM, Deshmukh A, Lerman A, Bossone E, Citro R, Ueyama T, Corrado D, Kurisu S, Ruschitzka F, Winchester D, Lyon AR, Omerovic E, Bax JJ, Meimoun P, Tarantini G, Rihal C, Y.-Hassan S, Migliore F, Horowitz JD, Shimokawa H, Lüscher TF, Templin C (2018). International expert consensus document on Takotsubo syndrome (part I): clinical characteristics, diagnostic criteria, and pathophysiology. Eur Heart J.

[24] Pelliccia F, Kaski JC, Crea F, Camici PG (2017). Pathophysiology of Takotsubo syndrome. Circulation..

[25] Manger WM (2006). An overview of pheochromocytoma: history, current concepts, vagaries, and diagnostic challenges. Ann N Y Acad Sci.

[26] Neumann HP, Young WF, Eng C (2019). Pheochromocytoma and paraganglioma. N Engl J Med.

[27] Manger WM (2005). The vagaries of pheochromocytomas. Am J Hypertens.

